# Methylphenidate Exposure Induces Dopamine Neuron Loss and Activation of Microglia in the Basal Ganglia of Mice

**DOI:** 10.1371/journal.pone.0033693

**Published:** 2012-03-21

**Authors:** Shankar Sadasivan, Brooks B. Pond, Amar K. Pani, Chunxu Qu, Yun Jiao, Richard J. Smeyne

**Affiliations:** 1 Department of Developmental Neurobiology, St. Jude Children's Research Hospital, Memphis, Tennessee, United States of America; 2 Department of Pharmaceutical Sciences, Bill Gatton College of Pharmacy, East Tennessee State University, Johnson City, Tennessee, United States of America; 3 Department of Information Sciences, St. Jude Children's Research Hospital, Memphis, Tennessee, United States of America; University of South Florida, United States of America

## Abstract

**Background:**

Methylphenidate (MPH) is a psychostimulant that exerts its pharmacological effects via preferential blockade of the dopamine transporter (DAT) and the norepinephrine transporter (NET), resulting in increased monoamine levels in the synapse. Clinically, methylphenidate is prescribed for the symptomatic treatment of ADHD and narcolepsy; although lately, there has been an increased incidence of its use in individuals not meeting the criteria for these disorders. MPH has also been misused as a “cognitive enhancer” and as an alternative to other psychostimulants. Here, we investigate whether chronic or acute administration of MPH in mice at either 1 mg/kg or 10 mg/kg, affects cell number and gene expression in the basal ganglia.

**Methodology/Principal Findings:**

Through the use of stereological counting methods, we observed a significant reduction (∼20%) in dopamine neuron numbers in the substantia nigra pars compacta (SNpc) following chronic administration of 10 mg/kg MPH. This dosage of MPH also induced a significant increase in the number of activated microglia in the SNpc. Additionally, exposure to either 1 mg/kg or 10 mg/kg MPH increased the sensitivity of SNpc dopaminergic neurons to the parkinsonian agent 1-methyl-4-phenyl-1,2,3,6-tetrahydropyridine (MPTP). Unbiased gene screening employing Affymetrix GeneChip® HT MG-430 PM revealed changes in 115 and 54 genes in the substantia nigra (SN) of mice exposed to 1 mg/kg and 10 mg/kg MPH doses, respectively. Decreases in the mRNA levels of *gdnf*, *dat1, vmat2*, and *th* in the substantia nigra (SN) were observed with both acute and chronic dosing of 10 mg/kg MPH. We also found an increase in mRNA levels of the pro-inflammatory genes *il-6* and *tnf-α* in the striatum, although these were seen only at an acute dose of 10 mg/kg and not following chronic dosing.

**Conclusion:**

Collectively, our results suggest that chronic MPH usage in mice at doses spanning the therapeutic range in humans, especially at prolonged higher doses, has long-term neurodegenerative consequences.

## Introduction

Methylphenidate (MPH; marketed under trade names Concerta® Metadate®, Methylin®, Ritalin®) is one of the most commonly prescribed stimulant medications for the symptomatic management of ADHD and narcolepsy [1], [2], [3]. MPH has been shown to have addictive potential, although it is not abused as frequently as cocaine [4]. Recent studies have detailed an increasing incidence of MPH abuse among young adults and college students in the United States, most likely for its purported non-therapeutic benefit of cognitive enhancement also called “neuroenhancement”. The Monitoring the Future Study (MTF) reported that 2.7% of high school students reported a non-therapeutic use of MPH while 1.9% of college students reported a similar non-medicinal usage [5], [6]

In both diagnosed ADHD and non-ADHD populations, MPH has been shown to increase scores on standardized tests [7], [8], as well as increase working memory [9] and thus, there have been calls for making it available as an “over the counter” (OTC) drug [10]. Despite the extensive use of this stimulant in ADHD and as well as for “off-label” use, few papers have been published regarding the long-term neurological consequences of MPH exposure in the CNS.

MPH is a Schedule II CNS stimulant that exerts its pharmacological effects via preferential blockade of the dopamine transporter (DAT) and norepinephrine transporter (NET), similar to that of cocaine [4]. This blockade results in a reduction of dopamine/norepinephrine uptake, leading to an increase in post-synaptic dopamine/norepinephrine levels [11], [12]. Thus, MPH usage leads to an acute increase in striatal dopamine levels [13]. In terms of neurological effects, dopamine has been shown to have a major modulatory effect in the developing brain on both neostriatal and cortical neurogenesis [14], [15]. Additionally, excess dopamine has been shown to be toxic both *in vitro* and *in vivo* due to the production of superoxide, hydrogen peroxide, and the dopamine quinone [16], [17], [18]. In fact, both acute and chronic treatment with MPH has been shown to result in superoxide production in the brain [19], [20], [21], [22], [23]. Free dopamine has also been shown to induce an inflammatory response in the brain characterized by an increase in cytokines and chemokines [24] that lead to an induction of microgliosis.

In this study, we investigate whether long-term administration of MPH in mice at two doses (1 mg/kg and 10 mg/kg) that reproduce the therapeutic window in humans (treatment of ADHD and recreational use/narcolepsy, respectively) [25], [26], [27] can induce changes in the basal ganglia. Specifically, we examined if acute or chronic administration of MPH altered SNpc dopamine neuron number and catecholamine levels in the striatum. Since excessive dopamine can induce oxidative stress and inflammation, we examined if MPH rendered the basal ganglia more sensitive to MPTP, an agent that has previously been shown to induce neuron damage in the SNpc.

## Results

### Chronic MPH administration affects SNpc DA neuron number

**Figure 1:**
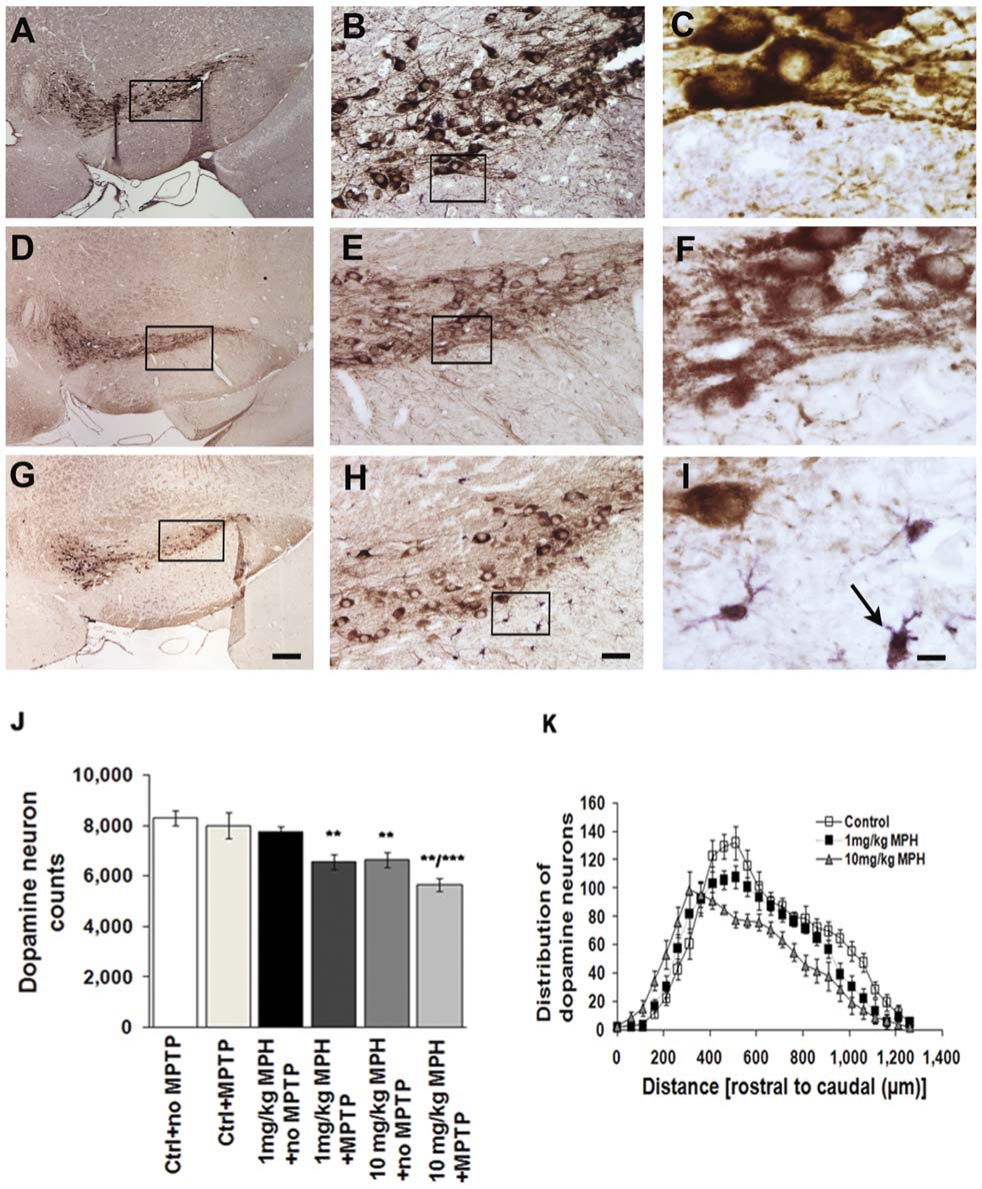
Chronic MPH administration affects SNpc dopamine neuron numbers and induces microgliosis. Representative images of the substantia nigra pars compacta (SNpc) from brains of animals treated with either saline (A–C), 1 mg/kg MPH (D–F) or 10 mg/kg MPH(G–I). The images presented are at 4×, 20× and 100×, respectively. The brain sections have been immunostained with anti-TH (brown) to identify dopaminergic neurons and anti-Iba-1 (purple) to identify microglia. (J) Stereological estimates of dopamine neuron number in substantia nigra pars compacta (SNpc) in animals administered saline (ctrl), saline+MPTP (ctrl+MPTP), 1 mg/kg MPH, 1 mg/kg MPH+MPTP, 10 mg/kg MPH and 10 mg/kg MPH+MPTP. Saline, 1 mg/kg MPH and 10 mg/kg were administered for 90 days following a one-week drug washout period before 4×20 mg/kg MPTP was injected (n = 10). (K) The distribution of dopamine neurons along the rostral-caudal axis in SNpc following chronic administration (90 days) of saline (control), 1 mg/kg MPH and 10 mg/kg MPH. **p≤0.01 compared to saline-treated controls; ***p≤0.001 10 mg/kg MPH compared to saline-control (ctrl), control+MPTP (ctrl+MPTP) and 1 mg/kg MPH+ no MPTP (n = 10). One-way ANOVA statistical test was performed to draw comparisons between the different groups followed by Bonferroni post-hoc tests. Scale bars (A,D,G), 200 µms; (B,E,H), 40 µms and (C,F,I), 8 µms.

We conducted a systematic stereological analysis of the SNpc in Swiss-Webster mice to determine if chronic exposure to saline, 1 mg/kg, or 10 mg/kg MPH for 90 days affected dopaminergic (DA) neuron number (Fig. 1A–I). While no change in SNpc DA neuron number was observed in animals treated with 1 mg/kg MPH, we did observe a 20% reduction of SNpc DA neurons in mice treated with 10 mg/kg MPH (Fig. 1J). The distribution of cell loss demonstrated that DA neurons towards the caudal end of the SN were more vulnerable to MPH effects while those residing in the more rostral end of this structure appeared unaffected (Fig. 1K).

### Chronic MPH exposure results in microglia activation in the SNpc

**Figure 2:**
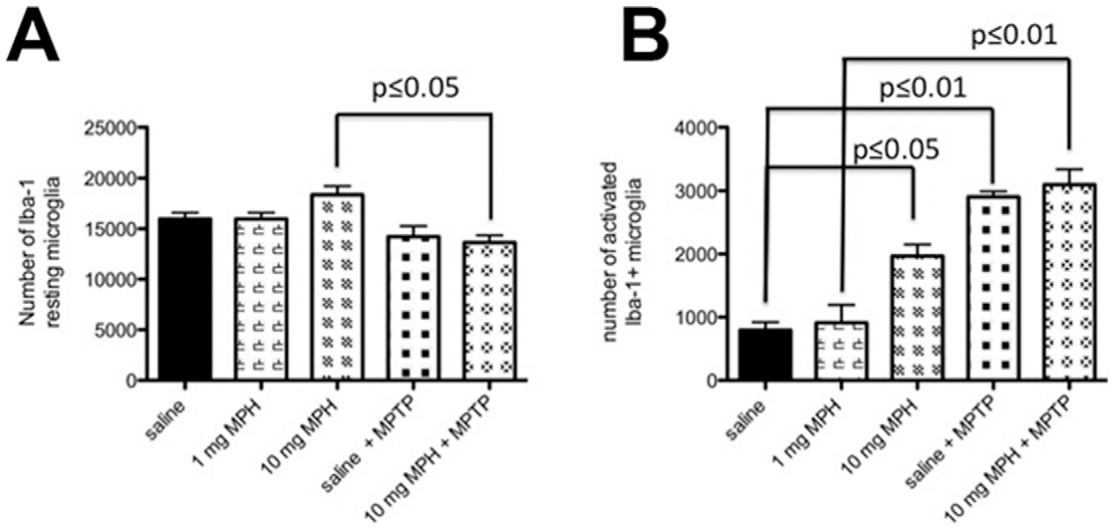
Chronic exposure to high dose MPH results in microglial cell activation in the SNpc. Stereological estimates of Iba-1 positive microglia cells in the SNpc (A) the total number of morphologically-resting microglia and (B) the total number of morphologically-activated microglia following chronic administration of either saline (ctrl), 1 mg/kg MPH, 10 mg/kg MPH and 10 mg/kg MPH+MPTP. (n = 5). One-way ANOVA statistical test was performed to draw comparisons between the different groups followed by Bonferroni post-hoc tests.

Since excess dopamine has been reported to induce oxidative stress and inflammation, we examined whether chronic administration of MPH could induce a pathological immunoligcal reaction in response to chronic MPH in the SNpc. We estimated the total number of Iba-1-positive microglia cells within the SNpc, and based upon morphology, determined the proportion of microglia in the resting and activated states. We observed that chronic administration of 10 mg/kg MPH did not affect the number of resting microglia (Fig. 2A), but did induce a significant increase in activated microglia (Fig. 2B). We did not observe any change in the number of resting or activated microglia after treatment with 1 mg/kg MPH (Fig. 2A,B).

### Dopamine and dopamine turnover affected following chronic MPH dosing

**Figure 3:**
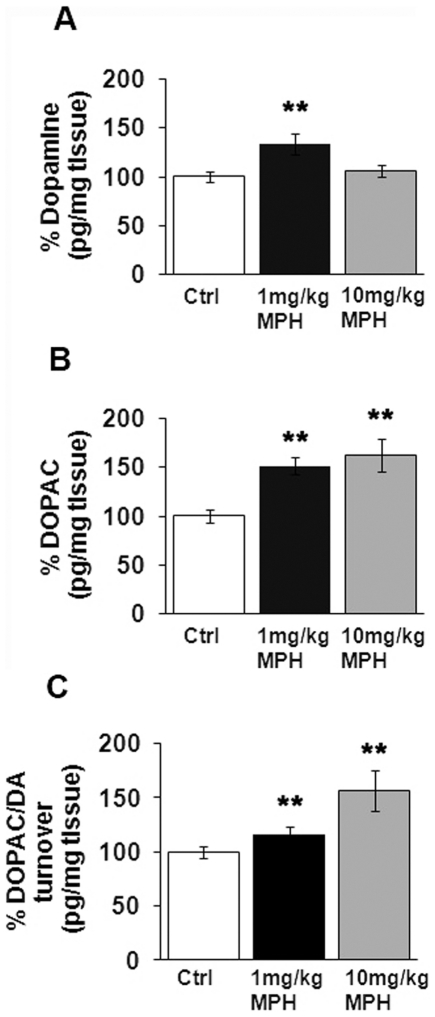
Chronic MPH dosing alters dopamine turnover in the striatum. Striata were microdissected from the brains of mice administered 90 days of saline (Ctrl), 1 mg/kg MPH or 10 mg/kg MPH and were processed for HPLC analyses. Total striatal levels of (A) dopamine and (B) the dopamine metabolite 3,4-dihydroxyphenylacetic acid (DOPAC) are presented as percentage of saline-treated controls (Ctrl). (C) Dopamine turnover is presented as the ratio of DOPAC/DA. *p≤0.01 compared to saline-controls (Ctrl) (n = 8). One-way ANOVA statistical test was performed to draw comparisons between the different groups followed by Bonferroni post-hoc tests.

In order to determine if long-term administration of MPH resulted in changes in total striatal dopamine levels or dopamine turnover, striata were microdissected 7 days after mice had been administered 90 days of saline, 1 mg/kg MPH, or 10 mg/kg MPH. We found that long-term administration of 1 mg/kg, but not 10 mg/kg MPH, induced a significant increase in total striatal dopamine compared to saline-injected controls (Fig. 3A). We also found a significant increase in the major dopamine metabolite 3,4-dihydroxyphenylacetic acid (DOPAC) at both 1 mg/kg and 10 mg/kg compared to saline-treated mice (Fig. 3B). However, a significant increase in dopamine turnover (DOPAC/DA) was observed only at 10 mg/kg MPH (Fig. 3C).

### Chronic MPH exposure sensitizes the SNpc to MPTP effects

Given that chronic administration of 10 mg/kg MPH-administration lowers SNpc DA neuron number, we examined whether chronic exposure to MPH increased the sensitivity of these neurons to the parkinsonian agent 1-methyl-4-phenyl-1,2,3,6-tetrahydropyridine (MPTP). We also examined if addition of MPTP after MPH altered the immunological microglial response.

In terms of SNpc dopaminergic neuron loss, we have previously shown that the Swiss-Webster strain is resistant to MPTP-induced neuron loss [28], [29]. Thus, if any SNpc neuron loss is observed, it can be inferred that this was due to previous exposure to MPH. We found that chronic administration of either 1 or 10 mg/kg MPH sensitizes SNpc DA neurons to the effects of MPTP compared with saline-injected controls. As shown in figure 1J, MPTP induces an approximately 20% increase in cell death in mice that received chronic administration of either 1 or 10 mg/kg MPH.

We also examined the microglial response to MPTP in these MPH-treated Swiss-Webster mice. Since we only observed an increase in activated microglia in mice chronically-administered 10 mg/kg MPH (Fig. 2B), we only examined the immune response to added MPTP in this condition. We found that mice administered 10 mg/kg MPH+MPTP exhibit a significant decrease in the number of resting microglia (Fig. 2A), with a concomitant rise in the number of activated microglia (Fig. 2B). This suggests that the MPTP potentiates the immune response to MPTP.

Although the increase in SNpc dopaminergic neuron loss was not large enough in and of itself to result in the onset of parkinsonism, this study does suggest that chronic administration of MPH has the potential to be a predisposing or contributing factor to disorders that lead to neurodegenerative disorders involving the dopaminergic system.

### Alterations in Gene Expression following Acute and Chronic MPH Exposure in SN

**Figure 4:**
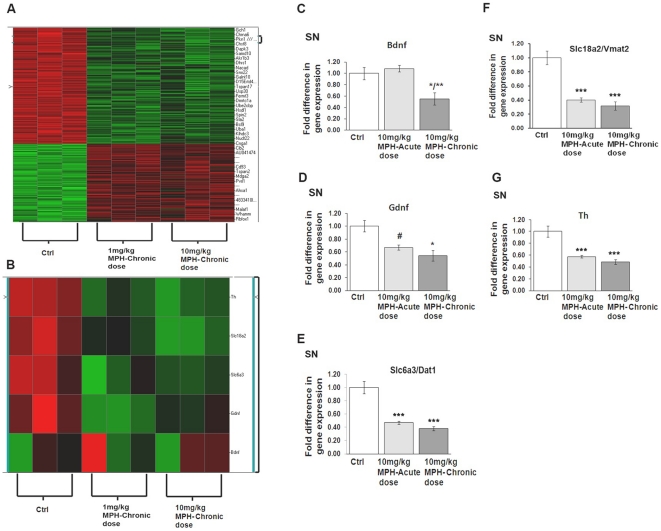
Acute and chronic administration of MPH alters gene expression in the substantia nigra (SN). (A) Heat map representation of gene expression changes following chronic administration of either 1 mg/kg MPH or 10 mg/kg MPH in the SN (n = 3). qPCR analysis demonstrating normalized fold-change expression of (B) *bdnf*, (C) *gdnf*, (D) *dat1(slc6a3)*, (E) *vmat2(slc18a2)* and (F) *th* mRNA in SN (n = 3). *p≤0.02 vs saline-controls (ctrl); **p≤0.02 vs saline-controls and 10 mg/kg MPH-acute dose; #p≤0.02 10 mg/kg MPH acute-dose vs saline-controls (ctrl). One-way ANOVA statistical test was performed to draw comparisons between the different groups followed by Bonferroni post-hoc tests.

In order to begin to identify the mechanism(s) underlying the MPH-induced decrease in SNpc DA neuron number and increases in CNS inflammation, we conducted an Affymetrix gene array study to identify SNpc mRNA changes that were induced in response to chronic administration of MPH. Using unsupervised hierarchical clustering analysis, probes were selected using a median absolute deviation score. Differentially expressed genes between each treatment condition and controls were derived using local-pooled-error test (LPE) with a FDR of 0.05 as the cut-off. We found a total of 115 genes and 54 genes out of 45,037 on the arrays whose expression were significantly different at the p≤0.05 at 1 and 10 mg/kg MPH doses, respectively, in the SNpc (Tables S1 and S2). Of these gene changes, 23 were common between the high and low MPH doses (Fig. 4A). Since the cellular changes, both in SNpc DA neuron number and microglia, were observed primarily at the 10 mg/kg MPH dose, we used qPCR to further examine and validate the expression of genes in animals exposed to only this dose. Specifically, we examined expression of genes associated with modulations in basal ganglia toxicity including brain derived neurotropic factor (*bdnf*), glial derived neurotropic factor (*gdnf*), tyrosine hydroxylase (*th*), the dopamine transporter DAT1 (*slc6a3*), and the vesicular monoamine transporter VMAT2 (*slc18a2*). We found significant reductions in mRNA expression in *gdnf*, *th*, *slc6a3*, and *slc18a2* after both acute and chronic administration of 10 mg/kg MPH, while *bdnf* was only reduced after chronic 10 mg/kg MPH (Fig. 4B–E).

### Evidence for inflammation associated with acute doses of MPH

**Figure 5:**
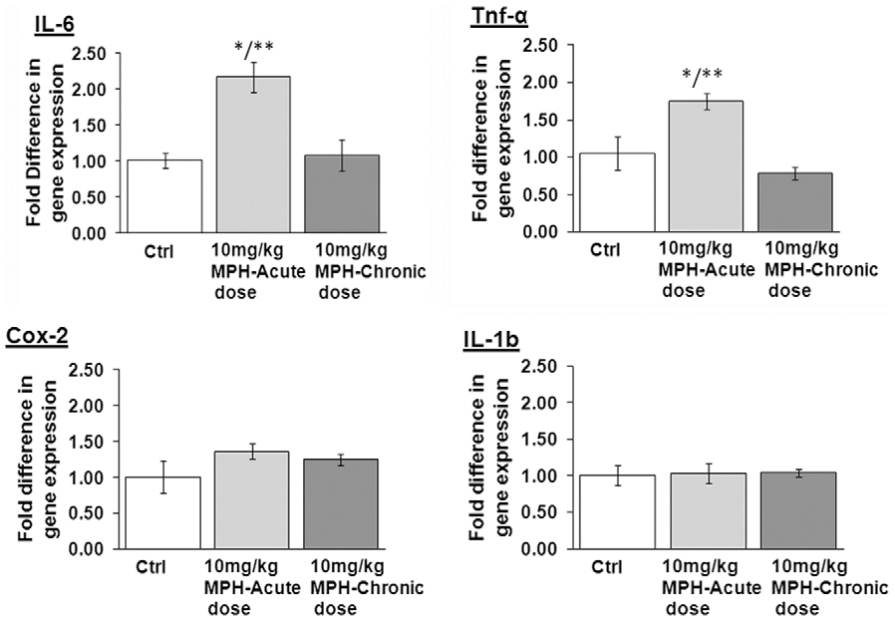
mRNA expression of pro-inflammatory genes following acute administration of 10 mg/kg MPH in the striatum. Fold change values in mRNA expression presented are normalized against saline controls, in the striatum. The genes probed for include (A) *il-6*, (B) *tnf-á*, (C) *cox2* and (D) *il-1b*. *p≤0.02 compared to saline-controls (ctrl); **p≤0.02 compared to 10 mg/kg MPH-chronic dose, (n = 3). One-way ANOVA statistical test was performed to draw comparisons between the different groups followed by Bonferroni post-hoc tests.

Due to the observed increase in the number of morphologically-activated SNpc microglia following administration of 10 mg/kg MPH, we investigated whether the expression of inflammatory genes, including *il-6*, *tnf-α*, *cox-2*, and *il-1b* were altered following chronic or acute dosing of 10 mg/kg MPH in both SN and its target, the striatum. We found significant increases in mRNA expression of the pro-inflammatory genes *tnf-α* and *il-6* in the striatum of animals administered a single dose of 10 mg/kg MPH compared saline-injected controls (Fig. 5A–D). No changes were seen in the expression of these genes in the SN.

## Discussion

The present study investigated the pathological effects of acute and chronic MPH in the basal ganglia using two different doses that span the therapeutic window of MPH use for ADHD and narcolepsy in humans (1 mg/kg and 10 mg/kg, respectively) [30], [31]. We demonstrate that chronic administration of 10 mg/kg MPH induces a small but significant loss of SNpc dopaminergic neurons. We also find that chronic exposure to both 1 mg/kg and 10 mg/kg MPH can sensitize SNpc dopamine neurons to a further oxidative stress. Though the complete mechanism for this sensitization of dopamine neurons is not well understood, our experiments suggest a combined effect of an increased inflammatory response with reduced levels of several trophic factors, including BDNF and GDNF.

Despite the extensive use of MPH in school aged and adult populations with ADHD (including a proportion that are improperly diagnosed with ADHD) [32], [33]) as well its use in general cognitive enhancement in non-ADHD individuals [10], only a few studies have investigated the neuropathological consequences of long-term MPH exposure. In this study, we used a 12-week MPH administration schedule that spans the developmental period in rodents and corresponds to the pre-adolescent through young adult period in humans, during which MPH is typically used [34].

MPH's mechanism of action is to increase the availability of extracellular DA and NE in the synaptic cleft through blockade of the dopamine transporter (DAT) and norepinephrine transporter (NET) [12], [35], [36]. In this study, we observed a significant increase in total dopamine levels in the striatum at 1 mg/kg MPH, a change that was not observed at 10 mg/kg MPH. Previous studies have also reported a similar increase in striatal dopamine levels at similar lower doses of MPH [37]. The observed lack of change in total dopamine concentrations at the higher dose might reflect a ceiling effect achieved due to chronic dosing of the drug, or it may be the result of a compensatory alteration in the production of dopamine that results from the observed 20% loss in dopamine neurons in the SNpc. In order to determine if this compensation is occurring, we measured the ratio of striatal dopamine to SNpc DA neurons. When examined as a ratio, both 1 and 10 mg/kg MPH treatment demonstrate a significant increase in the dopamine∶SNpc neuron ratios (150% in 1 mg/kg MPH and a 132% increase for mice treated with 10 mg/kg MPH), suggesting that either dose of MPH increases striatal dopamine, not just that of 1 mg/kg MPH.

It is well known that increased extracellular DA may be problematic. Oxidation of DA can produce both superoxide and hydrogen peroxide, which may then form hydroxyl radicals in the presence of certain metals [17]. Additionally, previous studies have indicated that DA can become neurotoxic following its oxidation to a DA quinone, which may then react with cellular thiols to form 5-S-glutathionyl DA and 5-S-cysteinyl DA [38]. The subsequent oxidation of 5-S-cysteinyl DA produces a number of neurotoxic compounds [17]. An increase in the free radical content in the basal ganglia has been shown to potentiate neurodegeneration [39], [40], [41].

In addition to a direct effect of MPH on the basal ganglia, we hypothesized that chronic MPH could increase sensitivity of SNpc dopamine neurons to a later oxidative stress exposure. MPH's mechanism of action- blockade of the DAT- is similar to that of cocaine [42] and results in an increase in extracellular dopamine, which has been shown to quickly form free radical adducts [43], [44]. Since increased free radical production has been shown to increase the sensitivity of SNpc neurons to environmental or administered xenobiotics [42], it is possible that long-term MPH could be a contributing etiological factor in a multi-hit hypothesis for induction of Parkinson's disease [45], [46], [47].

In this study, we administered an acute regimen of MPTP (4×20 mg/kg), an agent that is known to induce oxidative stress [48], [49], [50], to MPTP-resistant Swiss-Webster mice [28] treated with a chronic regimen of 1 or 10 mg/kg MPH. We found that chronic exposure to both 1 mg/kg and 10 mg/kg MPH increased the sensitivity of SNpc dopamine neurons to oxidative stress, based on a significantly increased SNpc dopamine neuron loss in mice administered MPH as compared to saline-treated control mice. Although the mechanism for this neuronal loss is unknown, a significant increase in MPH-induced activated microglia was observed; therefore, we hypothesize that an increase in free radical formation along with a concomitant neuroinflammatory response increases the sensitivity of the SNpc dopamine neurons to a later oxidative challenge. This conclusion is supported by a recent epidemiological study that showed that long-term amphetamine usage, which like MPH results in higher levels of striatal dopamine in the synaptic cleft, results in a significantly higher risk for developing Parkinson's disease [51].

In order to address the mechanism for increased sensitivity of dopamine neurons, we used an unbiased gene microarray analysis. A comparison of heat maps representative of relative mRNA expression (Figure 4A,B) shows a number of genes whose direction of expression change (±) was similar after chronic administration of 1 and 10 mg/kg. Gene Set Enrichment Analysis (GSEA) identified gene sets that were related to inflammation and cell damage and repair pathways. Using qPCR validation, we measured significant decreases in mRNA expression of the neurotrophins *bdnf* and *gdnf* in the SNpc after both acute and chronic dosing of 10 mg/kg MPH. We also found significant decreases in mRNA expression of genes involved in dopamine biosynthesis (tyrosine hydroxylase, *th*) and handling (dopamine transporter, (*slc6a3*) and the vesicular monoamine transporter (*slc18a2*)). These changes were observed following both acute and chronic doses of 10 mg/kg MPH in the SNpc. Previous reports have associated decreases in mRNA expression of *vmat2* and *dat1* with neurotoxicity in cases where pharmacotherapeutic agents that alter dopamine levels and neurodegenerative conditions, respectively [52], [53]. The observed downward changes in the mRNA message of *dat1* and *th* may also be due to the covalent modification by dopamine quinones leading to its translational inactivation [41], [54], [55].

Notably, our Affymetrix and qPCR studies also found that acute exposure to higher doses of MPH increased the expression of inflammatory genes in the striatum, including the pro-inflammatory genes *tnf-α* and *il-6*. This increase in the pro-inflammatory gene expression following a single acute dosage suggests that MPH does induce inflammation, and this is supported by our finding of increased numbers of both total and activated microglial cells in the SNpc. Surprisingly, we did not see an increase in inflammatory gene expression after chronic administration of MPH, although we did continue to observe an increased number of morphologically activated microglia. This suggests that sometime during the course of the chronic exposure to MPH, there might be a dampening (self-repressesion) of inflammatory gene expression. It is unknown at this time if the gene repression we observe after chronic treatment with MPH is permanent (longer than 90 days), or if it can at a later time be re-induced. If this is the case, then the activated microglia observed, have the potential to play a modulatory role in later inductions of oxidative stress that would affect the same brain systems. Alternatively, it is also possible that microglia that are activated do not have the ability to return to their morphologically pre-inflamed state, as other studies have shown evidence of microglia activation long after resolution of the initiating insult [56].

Taken together, our results suggest that chronic administration of methylphenidate in mice, at doses that approximate those at the higher therapeutic range in humans, results in a reduced expression of neurotrophic factors, increased neuroinflammation, and a small, but significant loss of SNpc dopamine neurons. These results can only be interpreted in the context on normal brain structure and function, and thus would have direct implications for the illicit/neurocognitive use of MPH. Since the underlying anatomy and biochemistry of ADHD has not been definitively characterized, our findings may or may not be generalizable to the vast majority of humans who are properly diagnosed with ADHD and are prescribed methylphenidate. Nevertheless, this work supports studies [51], [57], [58], [59] that demonstrate that drugs shown to increase the levels of dopamine in the synaptic cleft can contribute to degenerative changes in the basal ganglia.

## Materials and Methods

### Ethics Statement

All of the experimental procedures in the animals were performed in accordance with the NIH Guide for the Care and Use of Laboratory Animals. These studies were carried out under protocol number 322 and were approved by the St Jude Children's Research Hospital IACUC under the auspices of Animal Assurance Number: A3077-01, effective March 5, 2010–Dec. 31, 2013.

### Animal Handling and Treatment

Three week old Swiss Webster mice (Hsd:ND4, Harlan Laboratories) were acclimated in our animal facility for a period of a week and maintained on a 12 h light/dark cycle with *ad libitum* food and water. Starting at postnatal day PD28, mice were administered intraperitoneal (i.p.) injections of saline, 1 mg/kg, or 10 mg/kg methylphenidate hydrochloride (MPH, Cat # M2892 Sigma-Aldrich), once daily, 1 hour prior to the initiation of the animal's active phase (18:00 hrs). The doses of MPH used in this study were chosen based on previous studies in rodents suggesting that MPH doses of less than 5 mg/kg i.p. mirror those that are used in clinical practice [26], whereas recreational use of MPH or its use in the treatment of narcolepsy would be reflected by a dose of 10 mg/kg [27]. MPH injections were administered using a school week schedule (5/days/week), as this dosing paradigm is a recommended schedule for administration of MPH to eliminate the possibility of MPH to abrogate growth [60], [61]. At the end of 12 weeks, the animals were allowed a washout period of 7 days (except when noted) to ensure complete elimination of MPH from the CNS [62]. Subsequently, mice were either transcardially perfused with 3% paraformaldehyde for histological studies, or rapidly decapitated following an induction of deep anesthesia, after which individual brain regions were dissected and processed for mRNA isolation.

### Identification and Dissection of Brain Structures

Brain tissue from mice was dissected based on the following coordinates: SN (Bregma: −2.70 to −3.70) and striatum (Bregma: +0.14 to +1.26 mm) [63].

### Immunohistochemistry

After a one week drug washout period, Swiss-Webster mice were deeply anesthetized with an overdose of Avertin; following the loss of the deep tendon and corneal reflexes, mice were transcardially perfused with cold physiological saline followed by cold 3% paraformaldehyde. The perfused brains were processed for paraffin embedding. Brains were sectioned on the microtome at 10 µm thickness and mounted on polyionic slides (Superfrost-plus, Fisher Scientific). Deparaffinized sections were incubated with primary antibody for identification of dopamine neurons (mouse monoclonal anti-tyrosine hydroxylase, TH; Sigma-Aldrich;1∶500) or dopaminergic neurons and microglia (mouse monoclonal anti-tyrosine hydroxylase and rabbit polyclonal Iba-1 (Wako Chemicals; 1∶500)). The secondary antibodies included biotinylated mouse IgG (for TH, 1∶1000) or biotinylated rabbit IgG (for Iba-1, 1∶1000). Diaminobenzindine (DAB) or a VIP kit (Vector labs) reaction was used to yield a brown (TH) or a purple (Iba-1) color, respectively. All tissue sections were counter stained with the nissl stain Neutral Red for landmark identification.

### Quantification of SNpc DA neurons and Iba-1-positive microglia

DA neuron and Iba-1-positive microglial cell number in the SNpc were estimated using standard model-based stereological methods [64]. Briefly, for neuronal counts, brains were blocked and serially-sectioned at 10 µm from the rostral hippocampus to the cerebellar-midbrain junction. Serial sections were mounted 5 sections per slide onto polyionic slides. TH-positive neurons and TH-negative, Nissl-positive cells within the SNpc that had the characteristics of dopaminergic neurons were counted using a 40× objective (total magnification 400×). Specifically, neurons from both left and right sides of the SNpc within one section per slide (chosen randomly and then maintained throughout all sections, (i.e. the 3^rd^ section on each slide) were counted) [64].

Microglia were counted using the optical fractionator method [65] using Microbrightfield StereoInvestigator (MBF Biosciences, Williston, VT). Both Iba-1 resting and activated microglia were counted [66]. Stringent measures were adopted to classify Iba-1 positive microglia as resting or activated based on morphology based on the detailed description by Graeber and Streit [67]. Microglial cells would be deemed as resting if they contained a small oval Iba-1-positive cell body that averaged 3 microns in diameter with long slender processes. Microglia would be classified as activated when the cell body was slightly increased in size compared to resting microglia and had an irregular shape. Based on cell size of the counting particle in 12 micron (empirically measured) sections, we used a high NA lens and a total magnification of 1000× in which we were able to clearly define approximately 18 focal planes within our section (1 focal plane equals approximately 0.54 mm). The processes on the microglia were shorter and had thickened processes. All numbers are expressed as mean ± SEM.

### MPTP treatment

1-Methyl-4-phenyl-1,2,3,6-tetrahydropyridine (MPTP-HCl, Cat. #M0896, Sigma-Aldrich, St. Louis, MO) was dissolved in sterile saline to a final concentration of 5 mg/ml. Each animal was administered four intraperitoneal injections of 20 mg/kg MPTP-HCl, one every 2 hours for 8 hours. All mice were allowed to survive for one week after MPTP injections, a time when the MPTP-induced lesion was maximal [42].

### Microarray Analysis

Animals injected with either saline, 1 mg/kg MPH, or 10 mg/kg MPH for 3 months using a school-day schedule were allowed a 7 day drug wash period, after which animals were rapidly decapitated under deep anesthesia. The substantia nigra (SN) was rapidly dissected, flash frozen, and stored at −80°C. mRNA was isolated from SN in accordance with the protocol outlined in RNAqueous®-Micro kit (Ambion, Austin, TX) according to manufacturers recommendations. Technical procedures for microarray analysis, including quality control of mRNA, labeling, hybridization and scanning of the arrays were performed by the Hartwell Center for Bioinformatics & Biotechnology (HC) at St. Jude Children's Research Hospital (SJCRH) according to standard operating procedures for Affymetrix protocols (GeneChip® Expression Analysis manual, Affymetrix, Santa Clara CA, USA).

The mRNAs were profiled using Affymetrix HT MG-430 PM arrays. The array signals were normalized using Robust Multichip Average [68] and batch-effect of three replicates were corrected using ComBat [69]. The processed data were analyzed using linear models algorithm with Limma [70]. Differentially expressed genes between the treated and control samples were selected using FDR-corrected p-value of 0.01(q value of ≤0.05). All data are MIAME compliant, and the raw data have been deposited in a MIAME compliant database (GEOID: GSE33619).

### Validation of Microaray Data and Quantitative Analysis of mRNA levels

Dissected substantia nigra and striatum were homogenized and processed to yield mRNA in accordance with the protocol outlined in RNAqueous®-Micro kit (Ambion, Austin, TX). The isolated RNA was converted to cDNA using the High Capacity RNA to cDNA kit (Applied Biosystems, Carlsbad, CA). The cDNA was subsequently used for qPCR analysis using a Taqman assay (Applied Biosystems). The ribosomal 18S and beta-actin genes were used as the standardizing control gene. The final values have been expressed as 2^−ΔΔCt^ denoting fold-change in mRNA levels for each gene.

### Statistical Analysis

One-way ANOVAs with Bonferroni post-hoc tests were used to draw comparisons between treatment groups. Data was plotted as mean ± S.E.M. A value of p≤0.05 was considered significant.

## Supporting Information

Table S1 **Identification of differentially expressed genes (p≤0.05) in the substantia nigra comparing saline and 1 mg/kg MPH-treated mice.**
(XLS)

Table S2 **Identification of differentially expressed genes (p≤0.05) in the substantia nigra comparing saline and 10 mg/kg MPH-treated mice.**
(XLS)
