# Correction: Methylphenidate Exposure Induces Dopamine Neuron Loss and Activation of Microglia in the Basal Ganglia of Mice

**DOI:** 10.1371/annotation/c76da2c1-ccb8-4797-94c1-359d3ceceeda

**Published:** 2012-05-08

**Authors:** Shankar Sadasivan, Brooks B. Pond, Amar K. Pani, Chunxu Qu, Yun Jiao, Richard J. Smeyne

The equal contributions designation was inadvertently omitted. Brooks B. Pond and Shankar Sadasivan contributed equally to this manuscript. 

